# Reviewing the Potential Vectors and Hosts of African Swine Fever Virus Transmission in the United States

**DOI:** 10.1089/vbz.2018.2387

**Published:** 2019-06-26

**Authors:** Andrew J. Golnar, Estelle Martin, Jillian D. Wormington, Rebekah C. Kading, Pete D. Teel, Sarah A. Hamer, Gabriel L. Hamer

**Affiliations:** ^1^ Texas A&M AgriLife Research, Department of Entomology, Texas A&M University, College Station, Texas.; ^2^ Department of Veterinary Integrative Biosciences, Texas A&M University, College Station, Texas.; ^3^ Department of Microbiology, Immunology & Pathology, Colorado State University, Fort Collins, Colorado.

**Keywords:** Argasidae, vector competence, host competence, swine, African swine fever

## Abstract

African swine fever virus (ASFV) continues to threaten global animal health and agricultural biosecurity. Mitigating the establishment of ASFV in the United States (U.S.) is contingent on (1) the identification of arthropod vectors and vertebrate hosts that are capable of viral maintenance and transmission in the U.S. and (2) knowledge of vector-host associations that may permit transmission. We aggregated data on vector competence, host competence and tick–host associations by systematic review of published articles and collection records to identify species that may support the invasion of ASFV in the U.S. Three species of competent soft ticks occur in the U.S., *Ornithodoros coriaceus*, *Ornithodoros turicata*, and *Ornithodoros puertoricensis*, however, vector competence for the majority of soft ticks in the U.S. remains unknown. Three species of competent vertebrate hosts currently occur in the U.S.: domestic pigs (*Sus scrofa domesticus*), feral hogs (*Sus scrofa*), and common warthogs (*Phacochoerus africanus*). Hierarchical hazard categories based on vector competence, tick–host contact rates, and vector abundance were used to semiquantitatively rank U.S. soft tick species by their relative risk for contributing to ASFV transmission to identify which soft tick species are a priority for future studies. High-risk vector and host species identified in this study can be used to focus ASFV risk assessments in the U.S., guide targeted surveillance and control strategies, and proactively prepare for an ASFV incursion event. Results indicate *O. coriaceus*, *O. turicata*, and *O. puertoricensis* demonstrate the highest relative risk for contributing to ASFV transmission in the U.S., however, many gaps in knowledge exist preventing the full evaluation of at least 30 soft tick species in the U.S. Further study is required to identify soft tick vectors that interact with feral swine populations, elucidate vector competence, and further understand the biology of soft tick species.

## Introduction

African swine fever virus (ASFV) is a DNA virus (Family: *Asfarviridae*, Genus: *Asfivirus*) associated with severe epizootics and pathology in domestic pig populations. As such, viral outbreaks are accompanied with serious socioeconomic consequences and should be proactively managed (Fasina et al. 2012).

The virus was first described in Kenya in 1921 (Montgomery 1921) and has emerged in regions of Africa, Europe, the Caribbean, and South America facilitated by growing international trade networks and swill practices that expose swine to infection (Wooldridge et al. 2006, Costard et al. 2013, Roelandt et al. 2017). Although the virus has been extirpated at considerable cost from regions, including Brazil, Cuba, and Spain, the virus remains endemic in sub-Saharan Africa, Madagascar, and Sardinia (Italy) and in the last decade has caused multiple outbreaks in the Republic of Georgia, Azerbaijan, Armenia, Ukraine, Belarus, the Russian Federation, Lithuania, Poland, Estonia, Latvia, and most recently in the Luxembourg province of Belgium and the Anhui, Heilongjiang, Henan, Jilin, Liaoning, Jiangsu, Zhejiang provinces of China, and the Inner Mongolia Autonomous Region of China (Fernandez and Williams 1980, Arias and Sánchez-Vizcaíno 2002, Moura et al. 2010, Guinat et al. 2016a, Mur et al. 2016, Roelandt et al. 2017, ProMED-mail 2018).

The dynamics of ASFV transmission are complex and include several soft tick species of the *Ornithodoros* genus (Acari: Argasidae) and wild and domestic vertebrates from the family Suidae. As a DNA virus, ASFV is extremely persistent in its environment, such that contaminated secretions (blood, feces, urine, mucus) and fomites (vehicles, equipment) function as modes of transmission, in addition to vector-borne and aerosol transmission. Although vaccine technologies are in development, there is currently no vaccine or effective antiviral treatment. Disease management broadly aims to restrict animal movement, reduce contact between swine populations (domestic and wild), improve sanitation, and rapidly cull infected animals (Costard et al. 2009).

Although the roles for soft tick vectors and vertebrate hosts in disease maintanance, amplification, and spillover are diverse, a common feature of transmission is that the introduction of the virus to domestic populations results in severe epizootics, high case mortality, and disastrous economic consequences (Arias and Sánchez-Vizcaíno 2002, Moura et al. 2010, Fasina et al. 2012, Gogin et al. 2013, Brown and Bevins 2018). In the Russian Federation alone it is estimated that ASFV resulted in the loss of 800,000 pigs and between 833 million and 1.25 billion U.S. dollars since the virus was introduced in 2007 (United States Department of Agriculture 2017). As of October 6, 2018, just over 2 months following the initial report of ASFV in China (August 3, 2018), over 90,000 pigs have been culled in an effort to control the spread of ASFV (ProMED-mail 2018).

Due to the clear socioeconomic impacts of ASFV and a clear propensity to spread, ASFV is a pressing threat to global agricultural stability, especially for countries like China, which contains more than half of the world's pig population (Sanchez-Cordón et al. 2018). More than ever, it is important for countries with significant swine production to proactively prepare for an ASFV incursion event.

In endemic regions of Africa, ASFV is maintained in a sylvatic cycle among desert warthogs (*Phacochoerus aethiopicus*) and *Ornithodoros* tick species, including *Ornithodoros moubata* and *Ornithodoros porcinus porcinus*; however, bushpigs (*Potamochoerus porcus*) and giant forest hogs (*Hylochoerus meinertzhageni*) can also function as viral reservoirs. Although ASFV can be horizontally transmitted between swine (Guinat et al. 2016b, Davies et al. 2017), transmission from warthogs to domestic pigs is rare, suggesting soft tick vectors that inhabit pigsties, such as *Ornithodoros porcinus domesticus*, are important agents of viral spillover into domestic swine populations (Boinas et al. 2011).

Similarly, in regions of Europe, ASFV persists in a sylvatic cycle among wild boars (*Sus scrofa*) and the soft tick vector *Ornithodoros erraticus* (Jori and Bastos 2009). However, unlike the African system, recent reports posit that ASFV may persist among wild and domestic pig populations in the absence of soft tick vectors presumably through horizontal transmission (Pietschmann et al. 2016).

The ability to invade and persist without competent soft tick vectors further emphasizes the risk of ASFV to global swine populations and emphasizes a need to implement proactive disease management strategies (Guinat et al. 2014, 2016a, 2016b, Pietschmann et al. 2016). These data are particularly concerning to the agricultural security of the United States (U.S.), as the U.S. is one of the largest global swine industries and is now surrounded by an invasive population of over 6 million feral swine (*S. scrofa*) present in at least 35 states (Brown and Bevins 2018).

In anticipation of viral emergence in the U.S., agencies charged with mitigating viral invasion must focus on preventing introduction events, enhancing outbreak detection and preparing response strategies. The objective of this article is to systematically review and synthesize published vector competence data, vertebrate competence data, and tick–host association data to identify and rank tick and vertebrate species predicted to be important to the transmission of ASFV in the U.S. Results are intended to help prioritize future research on ASFV and protect health and economic stability in the U.S. by informing proactive prevention, detection, and response strategies.

## Methods

### Vector competence

Vector competence is the ability of an arthropod to acquire, support replication of, and transmit a pathogen to a susceptible vertebrate host. To identify competent vectors of ASFV, we retrieved articles written in the English language from the search engines Web of Science, NCBI's PubMed, and Science Direct with the search term “African swine fever virus.”

After reviewing abstracts, data from experimental studies in which vectors were exposed to ASFV were extracted and aggregated in a database for review. Data on arthropod species, virus strain, infectious dose, route of exposure, viremia, sample size, incubation period, temperature, infection rate, estimated transmission rates, viral multiplication, viral persistence, and transmission routes were recorded when available. We also recorded whether studies documented transtadial transmission (between arthropod life stages) and transovarial transmission (from adult female to offspring). Literature was identified and reviewed between March 2017 and March 2018. All soft tick species throughout the entire study are referred to using the authority designated by Guglielmone et al. (2010).

### Host competence

Host competence (also known as reservoir competence) is the assessment of an animal's ability to contribute to pathogen amplification and persistence and is a useful concept for judging the importance of different hosts to pathogen transmission (Komar et al. 2003, Golnar et al. 2014, Gervasi et al. 2015). In vector-borne disease systems, the role a vertebrate plays in amplifying a pathogen is often estimated based on the infectiousness of the host to a feeding vector and the frequency of contacts between infectious hosts and competent vectors (Kilpatrick et al. 2006, Hamer et al. 2009). Host infectiousness is often assumed to reflect the magnitude and duration of host parasitemia (defined broadly to include circulating titers of viral pathogens) observed during experimental infections (Komar et al. 2003).

In vector-borne disease systems, host parasitemia has been strongly associated with vector infection, vector dissemination, and vector transmission rates (Ewald 1987, Komar et al. 2003). The magnitude and duration of host parasitemia is also likely to provide a strong indicator of host infectiousness for pathogens transmitted between vertebrate hosts (Ewald 1987). Therefore, the review of vertebrate viremia profiles after ASFV infection provides a useful framework for assessing host competence.

Experimental transmission studies that documented viremic titers in vertebrate hosts written in English were identified and aggregated using similar search and inclusion criteria as those stated above. Studies were identified using Web of Science, PubMed, and Science Direct with the search term “African swine fever virus.” Titles and abstracts were reviewed to identify studies that exposed vertebrate hosts to ASFV and temporally quantify titers postinfection.

When available, vertebrate species, viral lineage, infectious dose, route of inoculation, and daily viremia postinfection were extracted and aggregated into a database for review. These data were used to understand the magnitude and duration of infection in different hosts by comparing viremia profiles over time. Although the viremic response in vertebrate hosts is expected to vary by viral strain, exposure dose, and route of inoculation, low sample sizes, nonstandardized methods, and the early termination of studies by euthanasia provided barriers to further statistical evaluation of these factors. In many cases, ASFV strains are genetically altered for various experimental questions, although genetically modified strains were excluded in this analysis, unaltered control ASFV isolates from the same studies were included.

### Vector–host association

The spatial distribution of soft tick species in the U.S. and contact rates between soft tick vectors and vertebrate hosts were estimated using a database provided by the U.S. National Tick Collection, Georgia Southern University, Statesboro, GA. Specifically, records between years 1891 and 2004 were aggregated based on the number of unique collections of each soft tick species that were recorded to be associated with different mammalian orders (Rodentia, Cingulata, Carnivora, Chiroptera, Lagomorpha, Artiodactyla, Perissodactyla, and Primates). Reptilian and Avian taxa were aggregated at the taxonomic resolution of class.

Although the number of ticks collected in each entry were variable (range from 1 to >2000 individuals), each entry for tick–host pairs was counted as one data point. It is important to note that soft tick species are often nidicolous and are frequently not collected directly from vertebrate hosts. For this analysis, we rely on the explicit assumption that all documented associations in the database are accurate.

### Soft tick risk index

To inform policy and future research directions, soft tick vectors in the U.S. were semiquantitatively ranked based on six hazard categories for which each tick species was assigned to the lowest (most severe) hazard level to which it qualified.

To participate in ASFV transmission, vectors must be competent, therefore vectors established to be competent were assigned a level 1 hazard (categorical value), which is considered the most severe. A vector's role in viral maintenance and amplification also depends on contact rates with competent hosts, therefore, tick association with the taxonomic family Suidae represents a level 2 hazard (continuous value) and tick association with the taxonomic order Artiodactyla is a level 3 hazard (continuous value). Because generalist feeding patterns on mammalian hosts may predict opportunistic contacts between soft tick vectors and swine, the number of mammalian hosts associated with a soft tick vector was defined as a level 4 hazard (continuous value).

Additionally, vector abundance is an important factor in assessing the relative risk of a tick vector for contributing to ASFV transmission in the U.S. As such, vector abundance was defined as a level 5 hazard (continuous value) and was estimated based on the number of U.S. counties, where ticks occur gathered from the U.S. National Tick Collection and the Global Biodiversity Information Facility (GBIF.org. 2018). Additionally, the number of collections reported to the U.S. National Tick Collection was utilized as an additional metric of vector abundance and was defined as a level 6 hazard (continuous value). The relative risk of a tick vector for contributing to ASFV transmission in the U.S. was determined by sorting the tick species by the defined hazard levels.

## Results

Literature search identified 170 studies through Web of Science, PubMed, and Science Direct that were published between 1966 and 2017. These articles covered topics, including ASFV epidemiology, viral ecology, disease management, vaccine development, controlled laboratory experiments, pathogenesis, mathematical biology, and subject reviews.

From these studies, 15 published studies were identified that provided data on vector competence (Wilkinson et al. 1981, McVicar 1984, Villeda et al. 1993, Ramiroibanez et al. 1995, Anderson et al. 1998, Argilaguet et al. 2012, Karalyan et al. 2012, Guinat et al. 2014, O'Donnell et al. 2015a, 2015b, 2016, Carlson et al. 2016, Sanford et al. 2016, Popescu et al. 2017, Basto et al. 2006a) and 27 studies provided data to assess host competence (Wilkinson and Donaldson 1977, Wilkinson et al. 1977, 1981, Thomson et al. 1980, McVicar 1984, Knudsen and Genovesi 1987, Genovesi et al. 1988, Villeda et al. 1993, Ramiroibanez et al. 1995, Anderson et al. 1998, Argilaguet et al. 2012, Ferreira et al. 2012, 2013, Karalyan et al. 2012, de Carvalho Ferreira et al. 2013, Guinat et al. 2014, Nieto-Pelegrin et al. 2015, O'Donnell et al. 2015a, 2015b, 2016, Pietschmann et al. 2015, Carlson et al. 2016, Reis et al. 2016, Sanford et al. 2016, Gallardo et al. 2017, Popescu et al. 2017, Post et al. 2017).

A total of 128 published articles identified during the literature search were not included because they did not provide quantitative data useful for evaluating vector or host competence (citations not provided).

### Vector competence

Globally, nine soft tick species (*Ornithodoros marocanus*, *Ornithodoros puertoricensis*, *Ornithodoros coriaceus*, *O. moubata porcinus*, *O. erraticus*, *O. moubata complex*, *Ornithodoros turicata*, *Ornithodoros savignyi*, *Ornithodoros parkeri*), four hard tick species (*Dermacentor reticulatus*, *Ixodes ricinus*, *Amblyomma americanum*, *Amblyomma mixtum*), and two insect species (*Triatoma gerstaeckeri* and *Stomoxys calcitrans*) have been evaluated for ASFV transmission competency.

Following engorgement on infectious blood, ASFV was detected in all vectors, except the hard ticks, *A. americanum* and *A. mixtum.* Infection prevalence was extremely variable by tick species, ranging between 0% and 100% within *multiple species* (Table 1).

**Table 1.** Summary of Published Transmission Experiments Exposing 15 Arthropod Species to African Swine Fever Virus

*Vector species*	*Endemic to USA*	*Sample size*	*Viral infections*	*Viral persistance (days)*	*Rate of infection*	*Transmission to pig*	*Viral replication*	*Transovarial transmission*	*ASFV induced mortality*	*Transtadial transmission*	*Sexual transmission*
*Ornithodoros coriaceus*	Yes	2003	Yes	502	83–93	Yes	—	No	Yes	Yes	—
*Ornithodoros puertoricensis*	Yes	4006	Yes	506	25–100	Yes	Yes	Yes	Yes	Yes	—
*Ornithodoros turicata*	Yes	—	Yes	23	—	Yes	—	—	—	—	—
*Amblyomma americanum*	Yes	120	No	—	—	No	—	No	—	—	—
*Amblyomma mixtum*	Yes	335	No	—	—	No	—	No	—	—	—
*Ornithodoros parkeri*	Yes	248	Yes	70	36	No	—	-	—	—	—
*Triatoma gerstaeckeri*	Yes	122	Yes	34	0–100	No	No	No	—	Yes	—
*Stomoxys calcitrans*	Yes	696	Yes	2	0–100	—	No	—	—	n/a	—
*Ornithodoros erraticus*	No	499	Yes	117	0–100	Yes	Yes	—	—	Yes	Yes
*Ornithodoros marocanus*	No	2273	Yes	655	28–100	Yes	No	—	Yes	Yes	—
*Ornithodoros moubata complex*	No	175	Yes	49	0–100	Yes	Yes	—	Yes	Yes	—
*Ornithodoros moubata porcinus*	No	51,096	Yes	239	0–100	Yes	Yes	Yes	Yes	Yes	Yes
*Ornithodoros savignyi*	No	337	Yes	9	0–80	Yes	No	No	—	Yes	—
*Dermacentor reticulatus*	No	56	Yes	56	0–100	—	—	—	—	—	—
*Ixodes ricinus*	No	171	Yes	42	67–100	—	—	—	—	—	—

Data on arthropod species, whether the arthropod is endemic in the United States, sample size, viral persistence (days), infection rate heterogeneity, vector competence, viral replication, transovarial transmission, parasite induced mortality, and transstadial transmission, are summarized from published studies (Plowright et al. 1970, 1974, Groocock et al. 1980, Mellor and Wilkinson 1985, Hess et al. 1987, Mellor et al. 1987, Endris et al. 1991, 1992, Endris and Hess 1992, Anderson et al. 1998, Kleiboeker et al. 1998, Rennie et al. 2000, Basto et al. 2006a, Ferreira et al. 2014, and Ribeiro et al. 2015). *Dashes* in cells indicate no data were available.

ASFV, African swine fever virus.

Evidence from vector competence studies demonstrate that eight taxa*—O. marocanus*, *O. puertoricensis*, *O. coriaceus*, *O. moubata porcinus*, *O. erraticus*, *O. moubata* complex, *O. turicata*, and *O. savignyi*—are competent vectors. Three of these species—*O. coriaceus*, *O. turicata*, and *O. puertoricensis*—exist in the U.S. The U.S. vectors *O. parkeri*, *T. gerstaeckeri*, *A. americanum,* and *A. mixtum* are considered incompetent hosts (Table 1). Infection by ASFV was detected in the U.S. vectors, *O. parkeri*, *T. gerstaeckeri*, and *S. calcitrans*, however, transmission of ASFV from *O. parkeri* and *T. gerstaeckeri* to a susceptible host failed. Transmission of ASFV from *S. calcitrans* to a susceptible host was not tested. Infection of ASFV was not detected in the hard ticks *A. americanum* and *A. mixtum* (Table 1).

Transmission studies demonstrate ASFV can be detected up to 655 days postinfection in *O. marocanus* and remain infectious for at least 588 days (Table 1). Viral replication of ASFV was reported in *O. puertoricensis*, *O. moubata porcinus*, and *O. moubata*, whereas a failure of replication of ASFV was reported in *O. marocanus*, *O. savignyi*, *T. gerstaeckeri*, and *S. calcitrans* (Table 1).

Transovarial transmission was documented in *O. puertoricensis* and *O. moubata porcinus*, but not in *O. coriaceus*, *O. savignyi*, *T. gerstaeckeri*, *A. americanum*, or *A. mixtum* (Table 1). Transstadial transmission was observed in *O. marocanus*, *O. puertoricensis*, *O. moubata*, *O. coriaceus*, *O. moubata porcinus*, *O. erraticus*, *O. moubata complex*, *O. savignyi*, and *T. gerstaeckeri* (Table 1). Sexual transmission between vectors was observed in *O. moubata porcinus* and *O. erraticus* (Table 1). Vector mortality due to ASFV infection was documented in *O. marocanus*, *O. puertoricensis*, and *O. coriaceus* (Table 1).

### Host competence

**FIG. 1.**
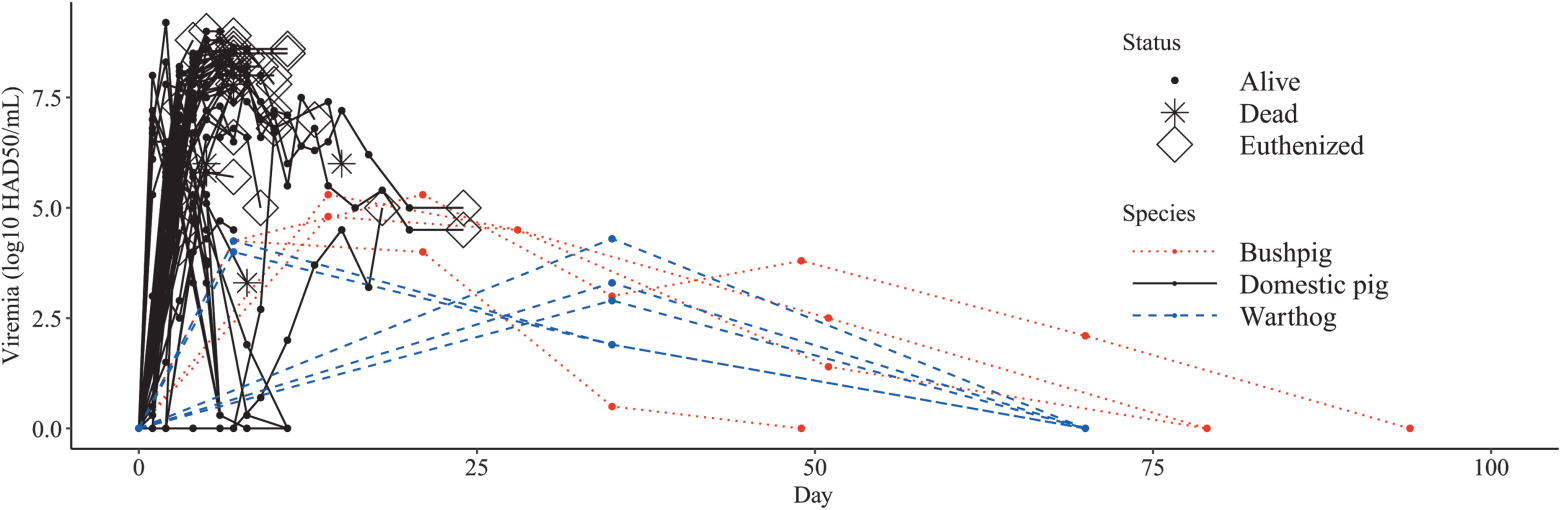
Viremia profile of vertebrates exposed to African swine fever virus. Viremia profiles of three vertebrate species exposed to African swine fever virus through intramuscular inoculation, intranasal infection, direct and indirect exposure between infectious pigs is visualized from 14 studies that documented viremia with HAD_50_ and HA (Wilkinson et al. 1981, McVicar 1984, Villeda et al. 1993, Ramiroibanez et al. 1995, Anderson et al. 1998, Argilaguet et al. 2012, Karalyan et al. 2012, Guinat et al. 2014, O'Donnell et al. 2015a, 2015b, 2016, Carlson et al. 2016, Sanford et al. 2016, Popescu et al. 2017). Studies quantified viral titers postinfection with HAD_50_ and HA, which are assumed to be equivalent measures of viral titer (Johnston et al. 1992). Vertebrate infectiousness depends on the magnitude of infection (*y*-axis) and the duration of infection (*x*-axis). Time series data on viremia titers ended when animals cleared infection (0.0 log_10_ HAD_50_/mL), animals died from infection (*), or were euthanized (⋄). HA, Hemagglutinin Assay; HAD_50_, Hemadsorption in 50% of inoculated cells.

Through methods of literature review, all species of vertebrate hosts exposed to ASFV were in the taxonomic family Suidae (bushpigs, warthogs, feral pigs, and domestic pigs). No studies included vertebrates outside of the Suidae family. Studies quantified viral titers postinfection with Tissue Culture Inoculation Dose 50 (TCID_50_), cytopathic effect (CPE), quantitative PCR, CPE, hemadsorption in 50% of inoculated cells (HAD_50_), and by hemagglutination assay (HA). Viremia profiles for 77 hosts (5 warthogs, 4 bushpigs, 68 domestic pigs) quantified by log_10_ HAD_50_/mL (Fig. 1) illustrate that domestic pigs produce the highest viremic titers (8.8 log_10_ HAD_50_/mL on day 6 P.I), followed by bushpigs (5.3 log_10_ HAD_50_/mL on day 21 P.I) and warthogs (4.3 log_10_ HAD_50_/mL day 35 P.I.).

Multiple warthogs were documented to have low viremia levels for up to 35 days P.I. (<4.3 log_10_ HAD_50_/mL) (Anderson et al. 1998) (Fig. 1). Virions were documented in a bushpig for up to 70 days (2.1 log_10_ HAD_50_/mL) (Anderson et al. 1998) (Fig. 1) and in domestic swine up to 40 days (4.2 1 log_10_ TCID_50_/mL) (not shown in Fig. 1) (Ferreira et al. 2012). It is important to note that animals infected with ASFV are often euthanized following IACUC-approved protocols suggesting that most estimates of viral persistence may be underestimations (Galindo-Cardiel et al. 2013).

### Tick–host association

The US National Tick Collection database contained records in the U.S. from 1891 to 2004 and documented the collection of 31,793 soft ticks, from 39 tick species, by more than 450 different individuals. Soft ticks were associated with swine in only one collection record: *O. coriaceus* ticks were documented to be associated with *S. scrofa* in Sonoma County, California in 1995. Four soft tick species were reported to be associated with nine different genera in the order Artiodactyla on 202 occasions.

**FIG. 2.**
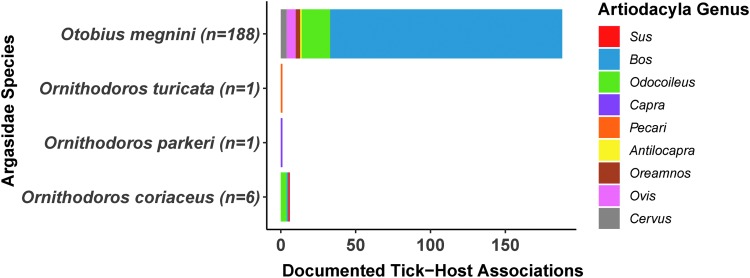
The association of Argasid ticks in the United States with vertebrate hosts in the Artiodactyla order by taxonomic genera. Soft tick (Argasidae) collection records across the United States from 1891 to 2004 were compiled from the U.S. National Tick Collection. Data represent the number of unique collections of each soft tick species that were associated with hosts from different Artiodactyla genera (*Antilocapra*, *Bos*, *Capra*, *Cervus*, *Odocoileus*, *Oreamnos*, *Ovis*, *Pecari*, *Sus*).

**FIG. 3.**
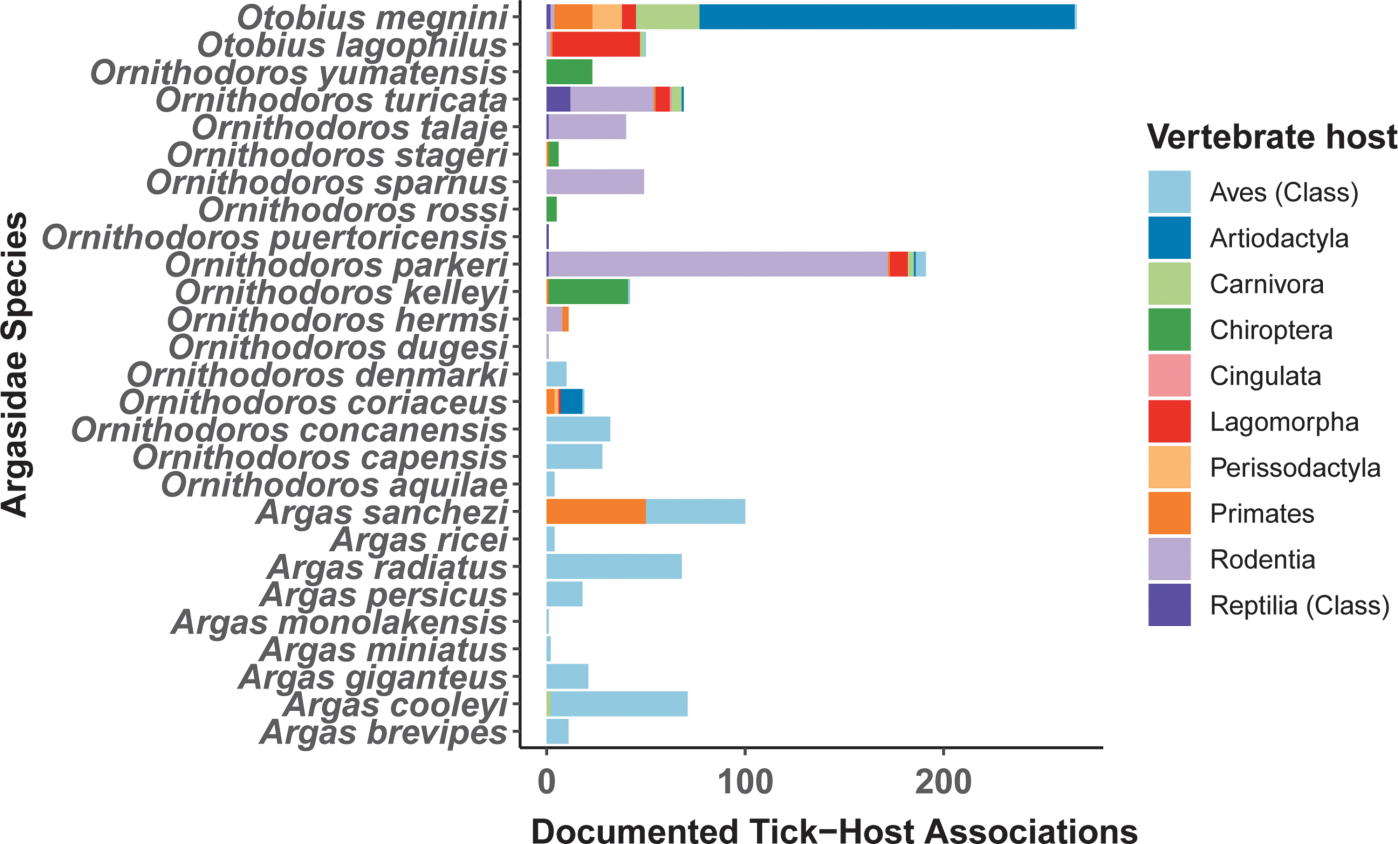
The association of Argasid ticks with vertebrate host orders in the United States. Soft tick (Argasidae) collection records across the United States from 1891 to 2004 were compiled from the U.S. National Tick Collection. Data represent the number of unique collections of each soft tick species that were associated with hosts from different mammalian orders (Rodentia, Cingulata, Carnivora, Chiroptera, Lagomorpha, Artiodactyla, Perissodactyla, and Primates). Vertebrate host taxa from the taxonomic groups of Aves and Reptilia are only documented at the resolution of Class.

Based on documented host associations, species associated with Artiodactyla were *Otobius megnini* (*n* = 190), *O. coriaceus* (*n* = 11), *O. turicata* (*n* = 1), and *O. parkeri.* (*n* = 1) (Fig. 2). Soft ticks that were associated with mammalian hosts include: *O. parkeri* (*n* = 204), *O. megnini* (*n* = 190), *O. turicata* (*n* = 62), *Ornithodoros kelleyi* (*n* = 55), *Otobius lagophilus* (50), *Ornithodoros sparnus* (*n* = 49), *Ornithodoros talaje* (*n* = 42), *Ornithodoros yumatensis* (*n* = 30), *O. coriaceus* (*n* = 19), *Ornithodoros stageri* (*n* = 17), *Ornithodoros hermsi* (*n* = 12), *Ornithodoros dyari* (*n* = 5), *Ornithodoros rossi* (*n* = 5), *Ornithodoros concanensis* (*n* = 4), *Argas cooleyi* (*n* = 5), *Argas sanchezi* (*n* = 2), *Ornithodoros dugesi* (*n* = 2), *Ornithodoros brasiliensis* (*n* = 1), *Ornithodoros hasei* (*n* = 1), and *Ornithodoros peropteryx* (*n* = 1) (Fig. 3).

Soft ticks from the genus *Argas* were generally associated with vertebrates from the Avian class (Fig. 3).

### Soft tick risk index

Vector competence and contact rates determined by host associations were used to rank which vectors would most likely be involved in ASFV transmission in the U.S.

*O. coriaceus* was ranked as the vector with the highest relative risk in the U.S. based on the hazard of vector competence and the hazardous associations with swine (Table 2). *O. turicata* was ranked as the vector with the second highest relative risk based on the hazard of vector competence and the hazardous associations with the vertebrate order Artiodactyla (Table 2, Figs. 2 and 3). *O. puertoricensis* was ranked as the vector with the third highest relative risk based on the hazard of vector competence (Table 2). *O. megnini* was ranked as the vector with the fourth highest relative risk based on their hazardous associations with hosts in the order Artiodactyla, however, the vector competence of this species remains unknown (Table 2).

**Table 2.** Soft Tick Vectors Ranked by Their Relative Risk to Contribute to African Swine Fever Virus Transmission in the United States

*Vector species*	*Vector competence*	*Vector*–*host associations in the United States by host species*	*Associated mammalian orders*	*U.S. counties with vector*	*Number of collections*	*Relative risk*
*Ornithodoros coriaceus*	Competent	*Bos taurus*, *Falco* sp., *Lepus* sp., *Sus scrofa*, *Equus ferus*, *Homo sapiens*, *Odocoileus hemionus*	Lagomorpha	31	20	1
			Artiodactyla			
			Chiroptera			
			Perissodactyla			
			Primates			
*Ornithodoros turicata*	Competent	*Canis latrans*, *Cynomys ludovicianus*, *H. sapiens*, *Pecari tajacu*, *Sciurus niger*, *Sylvilagus audubonii*, *Crotalus atrox*, *Crotalus horridus*, *Gopherus agassizii*, *Gopherus polyphemus*, *Neotoma micropus*, *Sylvilagus* sp., *Gopherus* sp., *Neotoma* sp., *Otospermophilus beecheyi*, *Lepus* sp., *Taxidea taxus*, *Dipodomys* sp., *Cynomys* sp.	Carnivora	78	87	2
			Rodentia			
			Primates			
			Artiodactyla			
			Cingulata			
*Ornithodoros puertoricensis*	Competent	*Ctenosaura acanthura*	—	1	1	3
*Otobius megnini*	Unknown	*A. americanum*, *Aspidoscelis gularis*, *Brachylagus idahoensis*, *Cnemidophorus martyris*, *Gallus gallus*, *H. sapiens*, *Lepus townsendii*, *Lepus* sp., *Marmota monax*, *Ovis canadensis*, *S. audubonii*, *Sylvilagus bachmani*, *Tamias* sp., *C. latrans*, *Lepus californicus*, *Oreamnos americanus*, *Cervus canadensis*, *Odocoileus* sp., *Ovis aries*, *Equus caballus*, *O. hemionus*, *Odocoileus virginianus*, *E. ferus*, *Felis catus*, *Canis lupus*, *B. taurus*	Artiodactyla	148	271	4
			Lagomorpha			
			Primates			
			Rodentia			
			Carnivora,			
			Perissodactyla			
*Ornithodoros parkeri*	Noncompetent	*C. latrans*, *Capra hircus*, *Cynomys gunnisoni*, *Dipodomys* sp., *H. sapiens*, *M. monax*, *Microtus longicaudus*, *Onychomys torridus*, *Otospermophilus variegatus*, *Petrochelidon pyrrhonota*, *T. taxus*, *Urocitellus washingtoni*, *Vulpes marcotis*, *Peromyscus maniculatus*, *Spermophilus* sp., *Sylvilagus* sp., *Urocitellus columbianus*, *Athene cunicularia*, *Cynomys leucurus*, *Lepus* sp., *Urocitellus richardsonii*, *O. beecheyi*, *Cynomys* sp.	Carnivora	70	210	5
			Artiodactyla			
			Rodentia			
			Primates			
			Lagomorpha			
*Otobius lagophilus*	Unknown	*Buteo swainsoni*, *C. lupus*, *Felis silvestris*, *H. sapiens*, *Spermophilus* sp., *Ochotona Unknown* sp., *Ochotona princeps*, *Sylvilagus* sp., *L. townsendii*, *L. californicus*, *Lepus* sp.	Carnivora	33	51	6
			Primates			
			Rodentia			
			Lagomorpha			
*Ornithodoros kelleyi*	Unknown	*Euderma maculatum*, *H. sapiens*, *Myotis subulatus*, *Myotis thysanodes*, *Nycticeius humeralis*, *Pipistrellus subflavus*, *Pipistrellus* sp., *Antrozous pallidus*, *Myotis lucifugus*, *Pipistrellus hesperus*, *Eptesicus fuscus*	Chiroptera	75	56	7
			Primates			
*Ornithodoros hermsi*	Unknown	*M. monax*, *Tamias* sp., *Urocitellus townsendii*, *Neotoma* sp., *Tamiasciurus hudsonicus*, *H. sapiens*	Rodentia	29	13	8
			Primates			
*Ornithodoros concanensis*	Unknown	*Buteo jamaicensis*, *Petrochelidon fulva*, *Falco sparverius*, *H. sapiens*, *P. pyrrhonota*, *Aquila chrysaetos*, *Falco mexicanus*	Chiroptera	34	39	9
			Primates			
*Ornithodoros stageri*	Unknown	*A. pallidus*, *H. sapiens*, *Myotis velifer*, *Tadarida* sp.,	Chiroptera	11	17	10
			Primates			
*Ornithodoros talaje*	Unknown	*Cynomys* sp., *Neotoma albigula*, *O. beecheyi*, *Reithrodontomys fulvescens*, *Sauromalus ater*, *T. hudsonicus*, *Spermophilus* sp., *Neotoma florida*, *Neotoma lepida*, *N. micropus*, *Dipodomys* sp., *Neotoma* sp.	Rodentia	37	43	11
*Argas sanchezi*	Unknown	*Callipepla gambelii*, *H. sapiens*, *Lophortyx* sp., *G. gallus*	Primates	36	50	12
*Ornithodoros yumatensis*	Unknown	*E. fuscus*, *M. thysanodes*, *Nyctinomus mexicanus*, *P. hesperus*, *P. subflavus*, *A. pallidus*, *Plecotus rafinesquii*, *Tadarida brasiliensis*, *Plecotus townsendii*, *M. velifer*	Chiroptera	21	30	13
*Ornithodoros sparnus*	Unknown	*Neotoma cinerea*, *Neotoma floridana* sp., *Peromyscus crinitus*, *Neotoma* sp., *P. maniculatus*, *N. lepida*	Rodentia	12	49	14
*Ornithodoros coprophilus*	Unknown	*—*	Chiroptera	8	12	15
*Ornithodoros dyeri*	Unknown	*—*	Chiroptera	6	5	16
*Ornithodoros rossi*	Unknown	*A. pallidus*, *E. fuscus*, *Leptonycteris nivalis*, *Macrotus californicus*, *Myotis evotis*	Chiroptera	5	5	17
*Ornithodoros dugesi*	Unknown	*N. micropus*	Rodentia	1	2	18
*Argas cooleyi*	Unknown	*Gymnogyps californianus*, *Strix varia*, *Tyto Alba*, *P. pyrrhonota*, *C. latrans and Vulpes macrotis*	—	44	72	19
*Argas radiatus*	Unknown	*Ardea herodias*, *B. swainsoni*, *Coragyps atratus*, *Egretta thula*, *Pelecanus occidentalis*, *Meleagris gallopavo*, *G. gallus*	—	39	68	20
*Argas persicus*	Unknown	*G. gallus*	—	22	19	21
*Carios capensis*	Unknown	*A. herodias*, *Bubulcus ibis*, *Haematopus palliatus*, *Nycticorax nycticorax*, *Platalea ajaja*, *Platalea* sp., *S. varia*, *Egretta rufescens*, *P. occidentalis*	—	11	29	22
*Argas giganteus*	Unknown	*A. cunicularia*, *Gymnorhinus cyanocephalus*, *Icteria virens*, *Megascops asio*, *Oreoscoptes montanus*, *Passerella iliaca*, *Pipilo aberti*, *Poecile gambeli*, *Salpinctes obsoletus*, *Callipepla gambeli*, *Pipilo fuscus*, *Toxostoma crissale*, *Zonotrichia leucophrys*, *Pipilo erythrophthalmus*	—	10	21	23
*Argas brevipes*	Unknown	*Baeolophus inornatus*, *Campylorhynchus brunneicapillus*, *Colaptes chrysoides*, *F. sparverius*, *Glaucidium brasilianum*, *Megascops kennicotti*, *M. asio*, *Melanerpes formicivorus*	—	7	12	24
*Carios denmarki*	Unknown	*Larus occidentalis*, *Phalacrocorax penicillatus*, *Sterna fuscata*, *Uria aalge*	—	8	11	25
*Argas ricei*	Unknown	*Cathartes aura*, *C. atratus*	—	4	4	26
*Argas miniatus*	Unknown	*G. gallus*	—	2	2	27
*Argas monolakensis*	Unknown	*Larus californicus*	—	2	1	28
*Ornithodoros aquilae*	Unknown	*A. chrysaetos*, *Buteo regalis*, *F. mexicanus*	—	1	4	29

Relative risk and ranking was determined by organizing tick species by the following hazard levels: vector competence (1), association with the taxonomic family Suidae (2), association with the taxonomic order Artiodactyla (3), number of associated mammalian host orders (4), geographic prevalence in the United States (5), and number of reported collections (6). *Dashes* in cells indicate that no data were available to infer hazard.

Based on no demonstrated hazard of ASFV vector competence or no hazardous associations with Artiodactyla hosts, 18 tick species were identified as low risk (Table 2). Besides *Ornithodoros parkeri*, the vector competence for all these species remains unknown. *O. parkeri* demonstrated no ability to transmit ASFV during a vector competence study and is therefore estimated to be low risk.

Finally, 16 soft tick species were classified to be of unknown risk. These species have no vector competence data and limited host association data (<10 documented associations with vertebrate hosts), which prevent hazard determination. Therefore, it is inconclusive whether these vectors may contact swine or contribute to ASFV amplification or maintenance (Table 2).

## Discussion

Animal health and agricultural security are significantly threatened by the propensity of ASFV to disperse and invade populations of swine worldwide (Costard et al. 2009, Roelandt et al. 2017, Brown and Bevins 2018). Accordingly, strategies to prevent and mitigate the invasion and establishment of ASFV in the U.S. must identify (1) which U.S. vectors and hosts are capable of contributing to viral transmission and maintenance and (2) understanding contact rates between competent vectors with competent hosts. In this study, we aggregate vector competence data, host competence data, tick prevalence, and tick–host association data to prioritize species that may play an important role in transmission and maintenance of ASFV should it arrive in the U.S.

Vector competence studies demonstrate that a variety of arthropods are susceptible to infection by ASFV. Mechanical transmission of the virus was observed in stable flies (*S. calcitrans*) (Mellor et al. 1987), but its role in endemic or epidemic transmission scenarios remains unknown. Furthermore, one experimental study demonstrated that ASFV may persist in kissing bugs (Family: Reduviidae, Subfamily: Triatominae) indicating host infection after accidental or the intentional ingestion of an infectious triatomine insect may be a potential mechanism of ASFV transmission, as observed in the Chagas disease system (Roellig et al. 2009, Pietschmann et al. 2015). However, whether domestic or wild swine ingest triatomine bugs or whether triatomine bugs are in contact with swine food sources needs further investigation (Pereira et al. 2010).

Overall, the capacity for arthropods to transmit the virus as biological vectors during a subsequent feeding event appears to be restricted to soft tick vectors in the genus *Ornithodoros*. In particular, only three species in the U.S. have demonstrated an ability to transmit ASFV: *O. coriaceus*, *O. puertoricensis*, and *O. turicata* (Table 1). However, transmission data for *O. coriaceus* and *O. turicata* were limited and experimental replicates should be completed to better understand transmission efficiency (Hess et al. 1987).

Further studies should focus on the unique aspects of soft tick biology and how this may influence ASFV transmission ecology. Soft ticks are known to survive for multiple decades as adults (Endris and Hess 1992), a unique trait that may afford ASFV a mechanism to persist in nature should vectors remain infectious. Furthermore, many soft tick vectors in the *Ornithodoros* genus feed multiple times through development, sometimes feeding as much as once every 3 days on multiple hosts, including other engorged ticks (Butler and Gibbs 1984). Experimental observations also demonstrate ASFV can persist for up to 588 days in some vector species, can be sexually transmitted between vectors, and vertically transmitted from adults to offspring (Endris and Hess 1992).

These biological characteristics highlight an incredible capacity for soft tick vectors to function as a viral reservoir and stress a need to understand soft tick biology in the U.S. to proactively identify and prevent mechanisms of ASFV establishment. Even though ASFV may be maintained through transovarial, sexual, and transtadial transmission, studies demonstrate infected *O. erraticus* and *O. moubata* colonies have cleared infections when reared on noninfectious blood suggesting there are limits to the long-term capacity of *Ornithodoros* ticks to function as viral reservoirs (Hess et al. 1989). Overall, the vector competence of most soft tick species in the U.S. remains unknown (Table 2) and additional vector competence studies are warranted. Future transmission experiments should focus on soft tick vectors that associate with competent hosts.

Numerous studies demonstrated that animals from the family Suidae are competent hosts, and there is a lack of experimental or epidemiological evidence to implicate any other mammalian taxa as amplification hosts. The experimental infection studies we reviewed show Suids are extremely susceptible to multiple strains of ASFV and can contract the virus through various modes of transmission, including direct tissue contact, fomites, aerosols, and vectors. Infection dynamics demonstrate that *S. scrofa* (domestic and feral pigs) produce relatively high viremic titers compared with sylvatic hosts found in endemic regions (bushpigs and warthogs) (Fig. 1). High viremia may contribute to pig-to-pig transmission in domestic swine operations; however, high contact rates in rearing operations likely play an important role (Wilkinson et al. 1977, Guinat et al. 2014).

Furthermore, it appears that Suids surviving clinical disease can support persistent viremic infections, in some cases for up to 70 days (Anderson et al. 1998, Ferreira et al. 2012). Assuming ASFV transmission is dependent on viremia levels, waning virus titers suggest that hosts become less infectious over time and, barring mechanisms of viral recrudescence or vertical transmission, persistently infected hosts may reach a point where they are no longer infectious. However, because most studies are terminated due to ethical or economic constraints, understanding the long-term dynamics of ASFV viremia continues to be a challenge.

A scarcity of biological and experimental data for most soft tick species in the U.S. makes it difficult to assess which vectors pose a risk for contributing to ASFV transmission. Knowledge of soft tick–host utilization and relative abundance can help identify which soft tick species are good candidates for vector competence studies. Contact rates are important in determining the potential contribution of organisms to pathogen transmission. Rates of contact between vertebrates and vectors can be difficult to assess in a natural setting, especially for *Ornithodoros* vectors that feed in a matter of minutes (Butler and Gibbs 1984). Molecular tools used to identify residual traces of host DNA in abdomens of vectors can be of use, but life history traits and trapping strategies have limited advancements in this area of soft tick ecology.

To overcome the fact that host association data remain underdeveloped for the majority of soft tick species in the U.S., we used metadata from the US National Tick Collection, which documents vertebrate hosts present in the area of collection to estimate tick–host contact. In this dataset, only a single soft tick species—*O. coriaceus—*was documented to be associated with swine in the U.S. Some tick vectors were associated with the sister taxa from the Artiodactyla order (Figs. 2, 3, and Table 2), including *O. megnini* with 190 records of being associated with Artiodactyla, suggesting this vector may persist in high abundance in domestic or peridomestic settings where livestock are held. In this context, high abundance may lead to incidental parasitism of domestic or peridomestic swine, although such parasitism has not been documented.

Overall, the lack of evidence demonstrating interactions between soft ticks and swine emphasizes a need to better understand which soft tick species interact with swine in the U.S. Prior studies demonstrate feral swine across Texas are infested with a variety of hard ticks, including with *A. americanum*, *A. mixtum*, *A. maculatum*, *Dermacentor albipictus*, *Dermacentor halli*, *Dermacentor variablis*, and *Ixodes scapularis* (Coombs and Springer 1974, Sanders et al. 2013). Although these efforts were quite extensive, infestations by Argasid ticks were not documented, including *O. megnini* despite the concentrated inspection of the ears where these parasites are known to commonly attach. Until intensive fieldwork across the U.S. updates our understanding of soft tick–swine interactions, we can utilize results generated by this synthesis to estimate the likelihood of tick–swine interactions.

Besides *O. coriaceus*, which has been documented to associate with swine, results from this analysis suggest *O. megnini*, *O. parkeri*, and *O. turicata* are the most likely soft tick species to associate with the family Suidae because of their association with the order Artiodactyla (Figs. 2 and 3). Soft ticks that associate with mammals (*O. lagophilus*, *O. kelleyi*, *O. hermsi*, *O. concanensi*, *O. stageri*, *O. talaje*, *and A. sanchezi*, *O. yumatensis*, *O. sparnus*, *A. coprophilus*, *O. dyeri*, *O. rossi*, *A. cooleyi*, *and O. dugesi*) are also potential candidates to utilize swine as hosts (Table 2).

## Conclusion

Our analysis highlights several gaps in knowledge for which additional research could enhance predictions of important hosts and vectors for ASFV and better inform proactive prevention and management strategies.

Overall, PCR is a useful diagnostic tool, however, molecular PCR-based methods for assessing virulence in experimental infections may result in overestimates of vector or host competence considering ASFV is a DNA virus capable of persisting as noninfectious nucleic acid (Basto et al. 2006b, Guinat et al. 2014). When possible, viral titrations should be performed in swine macrophage media and quantified by assessing 50% endpoints through hemadsorption to assess competence (Reed and Muench 1938, Plowright et al. 1968). Additionally, future vector transmission studies should make an effort to evaluate vector survivorship, vertical transmission, viral persistence, and alternative routes of transmission (*e.g.*, oral transmission) for soft tick species found in North America.

Similarly, host competence studies should try to evaluate dose-dependent transmission thresholds for vector transmission and horizontal transmission. Transmission thresholds and various routes of transmission appear to play an important role in ASFV ecology and epidemiology demonstrating the ability for ASFV to persist in a variety of ecological contexts (such as the absence of tick vectors) (Guinat et al. 2016a, 2016b, Pietschmann et al. 2016). However, knowledge on the impacts of mechanisms such as viral recrudescence or vertical transmission on viral maintenance remains underdeveloped (Anderson et al. 1998, Guinat et al. 2016a, Post et al. 2017).

Furthermore, even though evidence suggests hard ticks are not biological vectors of the virus (Ferreira et al. 2014), as a precautionary study, it may be necessary to test the vector competence of hard ticks associated with swine in the U.S. such as *A. maculatum*, *A. mixtum*, *Dermacentor variabilis*, or *Amblyomma tenellum* (Cohen et al. 2010, Corn et al. 2016). Finally, intensive field work across the U.S., where swine hosts persist, is necessary to incriminate species of soft ticks interacting with competent hosts in the U.S. and identify hot spots for potential ASFV establishment; this objective would be facilitated by enhanced bloodmeal analysis techniques to identify the vertebrate hosts upon which soft tick vectors have fed days, weeks, or years earlier.

No treatment or vaccine exists to prevent or combat ASFV infection, and subsequently, disease management is focused on intensive surveillance, restricting contact between wild and domestic swine, vector control, and policies that combat risky swill practices (Bellini et al. 2016, Halasa et al. 2016). In countries free of ASFV, such as the U.S., the importation of live pigs and pork products from infected areas is banned. However, if introduced to the U.S., the success of eradication relies on early detection, mass killing of infected animals, vector control, and proper disinfection. In this study, we demonstrate that *O. coriaceus*, *O. turicata*, and *O. puertoricensis* are high-risk vectors capable of contributing to ASFV transmission should the virus be introduced in the U.S. Accordingly, control and surveillance strategies in the U.S. can be tailored to the biology of these ticks.
